# *Aspergillus* spp. osteoarticular infections: an updated systematic review on the diagnosis, treatment and outcomes of 186 confirmed cases

**DOI:** 10.1093/mmy/myac052

**Published:** 2022-07-22

**Authors:** Andreas G Tsantes, Dimitrios V Papadopoulos, Eleftheria Markou, Konstantinos Zarokostas, Rozeta Sokou, Ioannis Trikoupis, Andreas F Mavrogenis, Dimitra Houhoula, Daniele Piovani, Stefanos Bonovas, Argirios E Tsantes, Athanasios Tsakris, Georgia Vrioni

**Affiliations:** Department of Microbiology, Medical School, National and Kapodistrian University of Athens, 15772 Athens, Greece; Department of Microbiology, ‘Saint Savvas’ Oncology Hospital, 11522 Athens, Greece; Department of Orthopaedic Surgery, Geisinger Medical Center, Danville, PA 17821, US; Department of Microbiology, University Hospital of Ioannina, Ioannina 45500, Greece; Department of Microbiology, ‘Saint Savvas’ Oncology Hospital, 11522 Athens, Greece; Neonatal Intensive Care Unit, “Agios Panteleimon” General Hospital of Nikea, Piraeus 18454, Greece; First Department of Orthopaedics, National and Kapodistrian University of Athens, School of Medicine, Athens 15772, Greece; First Department of Orthopaedics, National and Kapodistrian University of Athens, School of Medicine, Athens 15772, Greece; Laboratory of Haematology and Blood Bank Unit, “Attiko” Hospital, School of Medicine, National and Kapodistrian University of Athens, Athens 15772, Greece; Department of Biomedical Sciences, Humanitas University, 20090 Pieve MI, Milan, Italy; IRCCS Humanitas Research Hospital, Rozzano 20089 MI, Milan, Italy; Department of Biomedical Sciences, Humanitas University, 20090 Pieve MI, Milan, Italy; IRCCS Humanitas Research Hospital, Rozzano 20089 MI, Milan, Italy; Laboratory of Haematology and Blood Bank Unit, “Attiko” Hospital, School of Medicine, National and Kapodistrian University of Athens, Athens 15772, Greece; Department of Microbiology, Medical School, National and Kapodistrian University of Athens, 15772 Athens, Greece; Department of Microbiology, Medical School, National and Kapodistrian University of Athens, 15772 Athens, Greece

**Keywords:** *Aspergillus* spp, osteoarticular infection, diagnosis, management, antifungal agents

## Abstract

*Aspergillus* spp. osteoarticular infections are destructive opportunistic infections, while there is no clear consensus on their management. The purpose of this review is to investigate the current literature regarding *Aspergillus* spp. osteoarticular infections. An electronic search of the PubMed and Scopus databases was conducted considering studies that assessed osteoarticular infections from *Aspergillus* spp. We included only studies with biopsy proven documentation of positive cultures or histological findings for *Aspergillus* spp., and those with essential information for each case such as the anatomical location of the infection, the type of treatment (conservative, surgical, combination), the antifungal therapy, and the outcome. Overall, 148 studies from 1965 to 2021 including 186 patients were included in the review. One hundred and seven (57.5%) patients underwent surgical debridement in addition to antifungal therapy, while 79 (42.7%) patients were treated only conservatively. Complete infection resolution was reported in 107 (57.5%) patients, while partial resolution in 29 (15.5%) patients. Surgical debridement resulted in higher complete infection resolution rate compared to only antifungal therapy (70.0% vs. 40.5%, *P* < 0.001), while complete resolution rate was similar for antifungal monotherapy and combination/sequential therapy (58.3% vs. 54.5%; *P* = 0.76). Last, complete resolution rate was also similar for monotherapy with amphotericin B (58.1%) and voriconazole (58.6%; *P* = 0.95). The results of this study indicate that antifungal monotherapy has similar efficacy with combination/sequential therapy, while voriconazole has similar efficacy with amphotericin B. Moreover, surgical debridement of the infected focus results in better outcomes in terms of infection eradication compared to conservative treatment.

**Lay Summary:**

Antifungal monotherapy has similar efficacy with combination/sequential therapy, and voriconazole has similar efficacy with amphotericin B for the treatment *of Aspergillus* spp. osteoarticular infections, while surgical debridement of the infected focus improves the infection eradication rate.

## Introduction

Although fungal osteoarticular infections are rare opportunistic infections, their incidence is higher in certain population groups, such as in immunocompromised hosts. These destructive infections pose a challenging surgical problem that requires prolonged antifungal treatment. *Candida* genus is the most common fungal group that causes osteoarticular infections, followed by *Aspergillus* spp.^1^ Although A*spergillus* spp. usually infect the lower respiratory tract system causing invasive aspergillosis, they can also infect bones and joints with devastating sequelae.^2^ These pathogens have certain characteristics such as small conidia size that allows enhanced tissue penetration, while due to the abundance of negatively charged sialic acid on their surface they can strongly adhere to the host proteins.^3^ Their infectious potential is additionally increased by the presence of melanin in their surface that provides resistance to reactive oxygen species and phagocytosis. While there are several studies evaluating osteoarticular infections from *Candida* spp., there is only sparse information about osteoarticular infections from *Aspergillus* spp. Moreover, the available literature for osteoarticular infections from *Aspergillus* spp. is limited to small case series and case reports providing a lower quality level of evidence.^4,5^

The management of osteoarticular infections from *Aspergillus* spp. in terms of diagnosis and treatment requires a multidisciplinary approach and collaboration between infectious disease physicians, microbiologists, and orthopedic surgeons. The lack of sufficient data results in significant heterogeneity in the treatment of these infections in terms of the indicated antifungal agents, the antifungal regime (monotherapy vs. combination therapy/sequential therapy), the dose of the antifungal agents and the overall duration of the therapy. Moreover, the indication for surgical treatment is not as clear as in other bacterial osteoarticular infections.^6,7^

The purpose of this review is to investigate the current literature regarding the epidemiology, pathophysiology, diagnosis, and treatment of fungal osteoarticular infections from *Aspergillus* spp. We aimed to collect and report the available information only from studies with biopsy-proven osteoarticular aspergillosis that also provide sufficient information regarding the success or failure of well described treatment protocols in terms of eradicating these infections.

## Methods

### Search protocol/databases

A methodological protocol for systematic reviews based on the Preferred Reporting Items for Systematic Reviews and Meta-analyses (PRISMA) guidelines was followed in order to identify and evaluate studies that were assessing the topic of interest of this review. The review of the literature was performed between December 2021 and February 2022.

An electronic search in PubMed and Scopus databases was conducted until February 10, 2022 to identify eligible studies using a combination of the following terms: ‘aspergillus’, ‘aspergillosis’, ‘osseous’, ‘osteoarticular’, ‘musculoskeletal’, ‘bone’, ‘osteomyelitis’, ‘septic arthritis’, ‘orthopedic’, ‘spondylodiscitis’, ‘periprosthetic joint infections’. After an initial screening for eligibility by titles and abstracts of the retrieved articles, studies that were clearly irrelevant to our investigational target were excluded. The rest of the retrieved articles underwent full text review to evaluate whether they were meeting the inclusion criteria for this systematic review. The review process was independently performed by two investigators (EM and KZ), while any disagreement regarding the inclusion of the studies were resolved by a third author (AGT). Additionally, all the references cited in the articles that underwent full text review and all the references cited in previously published reviews were manually searched by two investigators (AGT and DVP) to identify any other studies, in order to minimize the possibility of missing out studies.

### Selection criteria

We considered studies assessing osteoarticular infections from *Aspergillus* spp. We evaluated randomized clinical trials, cohort studies (retrospective or prospective), and observational studies (case series or case reports) published in the English literature. Inclusion criteria included: (i) biopsy proven documentation of positive cultures or histological findings for *Aspergillus* spp. from a tissue sample taken from the infected osteoarticular location, and (ii) detailed report of essential information for each case: essential information was considered the anatomical location of the infection, the type of treatment (conservative, surgical, combination), the antifungal therapy (agents, monotherapy vs. combination therapy), and the outcome (complete resolution [defined as complete clinical improvement without any clinical signs of infection], partial resolution [defined as incomplete clinical improvement with partial resolution of the clinical signs of infection, with or without radiological findings of partial resolution of the infection], recurrence [defined as recurrent development of clinical signs of infection], death). Review studies, studies not involving humans, studies with suspected cases without documentation of positive culture or histological findings, and studies with inadequate information such as missing information about the treatment or outcome were excluded.

### Data extraction and data analysis

A standard excel sheet was used by two authors (EM and KZ) for an independent data extraction of the reported information from each included study. Data on patients’ demographics (age, gender) and underlying condition (corticosteroids, immunodeficiency, malignancy, etc.), microbiological data (*Aspergillus* species, coexisting infection from another pathogen), location (spine, cranial bones, thoracic ribs, etc.), route of infection (hematogenous, contiguous, direct inoculation), and clinical manifestations were extracted. Moreover, the type of biopsy and method for diagnosis (direct culture, histology etc.), the imaging findings and inflammatory markers (white blood cell (WBC) count, erythrocyte sentiment rate, C- reactive protein) were recorded. Last, data on the surgical treatment, antifungal therapy (monotherapy vs. combination therapy), antifungal regimen (agents, dose, duration), and outcome (complete resolution, partial resolution, recurrence, death from a non-infectious cause, death related to aspergillosis) were also extracted for each case.

Descriptive statistics were used to present the extracted data. Due to the high heterogeneity in extracted data, analysis of these data as a formal meta-analysis was not considered feasible, and only limited data analysis regarding the association between treatment and outcome was performed. Specifically, the infection resolution rate was compared between patients who received only antifungal therapy vs. patients who additionally underwent surgical debridement, between patients who received monotherapy vs. patients who received combination therapy, and between patients who received amphotericin B or voriconazole as monotherapy. Comparisons were performed using the chi square statistical test. Last, propensity score matching was performed using logistic regression models to reduce selection bias and adjust the comparison between patients who received amphotericin B and those who received voriconazole for certain confounding factors including age, gender, and underlying condition. The statistical analysis was performed using the Stata 15.0 software (Stata Corp., College Station, TX, USA), while a *P*-value lower than 0.05 indicated statistical significance.

## Results

### Study characteristics and patient demographics

**Figure 1.**
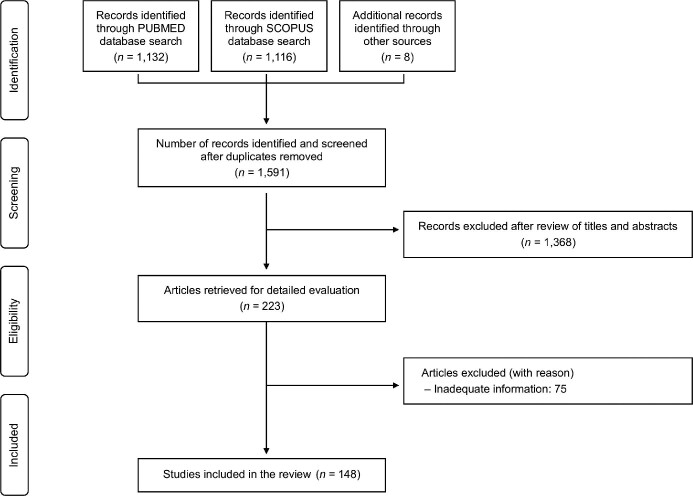
Flow diagram depicting the flow of information through the different phases of the systematic review.

Our search algorithm identified 1,132 records in PubMed database and 1,116 records in Scopus database, while 8 records were also identified through manual search of the reference lists of the articles that underwent full text review (Fig. 1). Following removal of duplicates from the retrieved articles, our search resulted in 1,591 records. After reviewing the abstracts/titles of these articles, 1,368 articles were considered clearly irrelevant to the topic of interest and were excluded, mainly because they were evaluating different pathogens, or they were not on humans. In a following step, 223 articles underwent detailed evaluation through full text review, and 75 of these were excluded because they did not provide sufficient information based on the inclusion criteria of our review protocol. Therefore, 148 studies including 186 patients met the eligibility criteria of this review and were included.^8–155^ The included studies were conducted over a period from 1965 to 2021. All included studies were case reports or small case series with up to 7 cases, while there were no cohort comparative studies. In particular, among the 148 studies, there were 130 studies reporting just one case, 11 studies reporting 2 cases, 2 studies reporting 3 cases, 2 studies reporting 4 cases, 1 study reporting 5 cases, 1 study reporting 6 cases, and 1 study reporting 7 cases.

The median age of the 186 patients was 44 (Interquartile Range [IQR]: 20–60) years, while osteoarticular aspergillosis was more common in men (125 males, 67.2%). There were 30 (16.1%) pediatric cases (<14 year-old) among the included cases. The most common underlying condition in these patients was chronic granulomatous disease (*n* = 35, 18.8%), a genetic disorder characterized by immunodeficiency, followed by hematologic neoplasia (*n* = 30, 16.1%), other causes of immunodeficiency (immunosuppression therapy or other immunodeficiency disorder; *n* = 23, 12.3%) and diabetes (*n* = 23, 12.3%). Moreover, 33 (17.7%) patients were on corticosteroids when they developed osteoarticular infection from *Aspergillus* spp., while 11 (5.9%) patients were IV drug users. For 21 patients (11.2%) relevant information about presence of any underlying condition was missing. The demographics and comorbidities of the study population are summarized in Table 1.

**Table 1.** Demographics of the study population.

Variables	Patients (*n* = 186)
Age (years)	40.9 ± 21.4, 44 (20–60)
Gender (male)	125 (67.2)
Comorbidities/Underlying condition Chronic granulomatous disease Hematologic neoplasia Immunodeficiency/immunosuppression therapy Diabetes Tuberculosis Other IV drug users None Not mentioned	35 (18.8) 30 (16.1) 23 (12.3) 23 (12.3) 14 (7.5) 15 (8.0) 11 (5.9) 14 (7.5) 21 (11.2)
Corticosteroids	33 (17.7)

Data are presented as means ± SD, medians and IQR, or as absolute frequencies (percentages) when appropriate.

### Location and route of infection

Overall, 192 infected foci were identified in the 186 included patients. Osteoarticular aspergillosis in two different anatomic locations was evident in 6 (3.2%) patients. Aspergillosis was located in spine causing spondylodiscitis with or without abscesses in 100 out of the 192 infected locations (52.0%), while the second most common location included the osseous structures of the thoracic cavity including the ribs and the sternum (18.2%, **Supplementary Table 1**). Other common locations for these infections included the long bones of lower extremities (*n* = 9, 4.6%) and the bones of the foot (*n* = 6, 3.1%), while the cranial bones (*n* = 9, 4.6%) and the maxilla/mandible (*n* = 5, 2.6%) were also commonly infected. Last, septic arthritis due to aspergillosis was evident in 22 (11.3%) patients, with the knee being the most commonly infected joint (*n* = 10, 5.2%).

The route of infections varied based on the location of the infection. Overall, the most common route of infection overall was contiguous spread (*n* = 72,37.5%) followed by direct inoculation after surgery or trauma in 64 cases (33.3%; 25 cases following surgery and 39 cases following trauma) and hematogenous spread in 56 cases (29.1%, **Supplementary Table 1**). Hematogenous spread was the most common route for spinal (*n* = 42, 42.0%) and cranial infections (*n* = 4, 44.3%), while for infections in the osseous structures of the thoracic cavity the most common route was contiguous spread (*n* = 25, 71.4%). Conversely, direct inoculation was the most common cause for aspergillosis in joints (*n* = 14, 63.6%).

Pulmonary involvement was evident in 41 patients (22.0%) with osteoarticular infection from *Aspergillus* spp. Among these 41 patients, the osteoarticular infection was located in the osseous structures of the thoracic cavity in 25 patients, in the spine in 13 patients, and in the cranial bones in 3 patients.

### Microbiology and markers of inflammation

**Figure 2.**
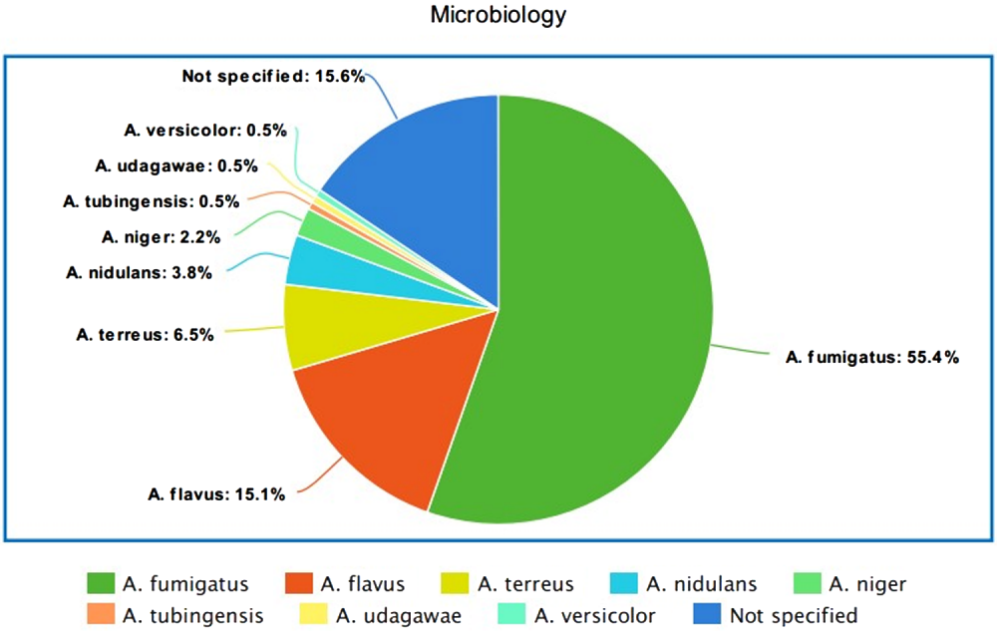
Microbiology of *Aspergillus* species. Mycological identification was performed through direct culture, histologic examination, and PCR analysis.

In the majority of patients the fungal infections were caused by *A. fumigatus* (*n* = 103, 55.3%), while other common *Aspergillus* spp. causing bone and joint infections included *A. flavus* in 28 patients (15%), *A. terreus* in 12 patients (6.4%), and *A. nidulans* in 7 patients (3.7%, Fig. 2). In 29 cases (15.6%) the pathogen was not specified. Interestingly, concomitant bacterial infections were reported in 13 patients (6.9%), with the most common co-cultured pathogens being Staphylococcus spp. (*n* = 7, 3.7%) followed by Enterobacteriaceae (*n* = 4, 2.1%) and Pseudomonas aeruginosa (*n* = 2, 1.0%).

In 92 (49.4%) patients *Aspergillus* osteomyelitis was diagnosed by positive cultures, while in 84 (45.1%) *Aspergillus* spp. were detected by both cultures and histological examination. In 5 (2.6%) patients the diagnosis was established by positive cultures and positive Polymerase Chain Reaction (PCR), and in 4 (2.1%) patients aspergillosis was confirmed by positive cultures and elevated serum galactomannan index, a fungal cell wall component. Regarding the 6 patients with positive PCR for *Aspergillus* spp., PCR analysis was performed on tissue sample in 4 patients, while in 2 patients the source of the specimen for the PCR analysis was not reported. Moreover, in 1 (0.5%) patient aspergillosis was diagnosed by positive cultures, histology examination, and positive serological findings (elevated serum galactomannan index, Table 2).

**Table 2.** Diagnostic studies and markers of inflammation.

Studies	Patients (*n* = 186)
Direct culture	92 (49.4)
Histology and direct culture	84 (45.1)
Direct culture and PCR	5 (2.6)
Direct cultures and serology	4 (2.1)
Direct cultures, histology and PCR	1 (0.5)
WBC count (× 10^3^/ml)	9.3 ± 4.5, 8.7 (6.0–12.5)
*Abnormal WBC count (>12 × 10^3^/ml)	61 (32.7)
CRP (mg/L)	78.4 ± 69.8, 52.0 (24.0–105.0)
*Abnormal CRP (>10 mg/L)	168 (90.3)

Data are presented as means ± SD, medians and IQR, or as absolute frequencies (percentages) when appropriate.

*Abbreviations:* PCR, Polymerase Chain Reaction; WBC, White Blood Cell; ESR, Erythrocyte Sedimentation Rate; CRP, C Reactive Protein.

*WBC count and CRP levels were missing for 25 and 18 patients, respectively.

Regarding the markers of inflammation, the median WBC count was 8.700 (IQR: 6.000–12.500)/ml, while the median C Reactive Protein (CRP) was 52 (IQR: 24–105) mg/L. Interestingly, WBC count was elevated (> 12.000/ml) only in 61 (32.7%) patients with osteoarticular aspergillosis, as opposed to CRP levels which were abnormal in 168 patients (90.3%; Table 2). Information about WBC count and CRP levels were missing for 25 patients (13.4%) and 18 patients (9.6%), respectively. In 2 immunocompromised patients (1.0%) severe neutropenia (<500 cells/ml) was also reported.

### Antifungal agents, protocols, and treatment outcomes

The antifungal regimens, the duration of treatment, and the rates of complete resolution are summarized in Table 3. Overall, 107 (57.5%) patients underwent surgical debridement in addition to antifungal therapy, while 79 (42.5%) patients were treated conservatively and received antifungal therapy. Although in most studies the indications for surgical treatment were not reported, the authors of some studies reported failed conservative treatment and clinical deterioration as surgical indications. Moreover, the location of the infection was associated with the decision for surgical treatment since all patients with septic arthritis (22 patients) and most patients with infection in lower/upper extremities (16 patients) underwent surgical treatment, as opposed to patients with infections in other locations such as in spine, who were mostly treated conservatively. The duration of antifungal treatment was similar between patients who were treated surgically and those who were treated conservatively (medians: 145 days vs. 120 days, *P* = 0.79).

**Table 3.** Treatment protocols and rates of complete infection resolution.

Treatment modalities	Patients (*n* = 186)	Duration (days)	Complete resolution
Total	186 (100)	155.5 ± 110.9, 126 (63–190)	107 (57.5)
Amphotericin B monotherapy	55 (29.5)	62.1 ± 40.1, 56 (30–70)	32 (58.1)
Voriconazole monotherapy	46 (24.7)	158.2 ± 94.2, 140 (97–180)	27 (58.6)
Amphotericin B + Itraconazole	28 (15.0)	164.9 ± 143.0, 112 (70–201)	16 (57.1)
Itraconazole monotherapy	16 (8.6)	204.0 ± 125.2, 180 (90–328)	8 (50.0)
Amphotericin B + 5-Fluorocytosine	15 (8.0)	66.0 ± 22.6, 63 (45–90)	6 (40.0)
Amphotericin B + Voriconazole	8 (4.3)	158.4 ± 90.4, 144 (105–204)	3 (37.5)
Other antifungal regimes	34 (18.2)	120.9 ± 50.4, 110 (80–150)	15 (44.1)
Surgical debridement	107 (57.5)	155.3 ± 104.6, 145 (60–194)	75 (70.0)
Conservative treatment	79 (42.5)	155.9 ± 80.1, 120 (20–180)	32 (40.5)

Data are presented as means ± SD, medians and IQR, or as absolute frequencies (percentages) when appropriate.

Regarding the antifungal treatment, 120 (64.5%) patients received antifungal monotherapy while the rest 66 (34.5%) received a combination/sequential therapy. Fifty-five (29.5%) patients received amphotericin B (either liposomal or deoxycholate) as monotherapy, 46 (24.7%) patients received voriconazole as monotherapy, and 16 (8.6%) patients received itraconazole as monotherapy. Moreover, 28 (15.0%) patients received a combination/sequential therapy with amphotericin B and itraconazole, 15 (8.0%) patients received a combination/sequential therapy with amphotericin B and 5-Fluorocytosine, and 8 (4.3%) received a combination/sequential therapy with amphotericin B and voriconazole. Overall, amphotericin B was administered in 114 (61.2%) patients (amphotericin B deoxycholate in 28 patients, liposomal amphotericin B in 56 patients, and not defined in 30 patients). Voriconazole was the second most common antifungal agent administered in 61 (32.7%) patients with a median daily dose of 400 (380–460) mg, and itraconazole was the third most commonly used antifungal agent, administered in 48 (25.8%) patients with median daily dose of 400 (IQR: 400–400) mg (**Supplementary Table 2**).

Regarding the outcome, complete resolution of the infection was reported in 107 (57.5%) patients, while partial resolution was reported in 29 (15.5%) patients and recurrence of the infection in 17 (9.1%) patients (Table 4). The median time of recurrence after the end of the antifungal treatment was 4.2 (IQR: 2.1– 6.9) months. The overall mortality rate was 16.1% (*n* = 30). In most deceased patients (*n* = 27, 14.5%) their death was not related to aspergillosis, while death related to aspergillosis was reported in 3 (1.6%) patients. Complete resolution of the infection was more common in patients who were treated surgically (*n* = 75, 70.0%) in addition to antifungal therapy, compared to those who received only antifungal therapy (*n* = 32, 40.5%, *P* < 0.001). Conversely, patients who received antifungal monotherapy and patients who received combination/sequential antifungal therapy had similar rate of complete infection resolution (*n* = 70, 58.3% vs. *n* = 36, 54.5%; *P* = 0.76). Last, the rate of complete infection resolution was also similar between patients who received amphotericin B as monotherapy (*n* = 32, 58.1%) and patients who received voriconazole as monotherapy (*n* = 27, 58.6%; *P* = 0.95). The rate of complete infection resolution was also similar for amphotericin B and voriconazole after propensity score matching adjusted for age, gender, and underlying condition (Odds Ratio: 1.14, 95% Confidence Interval: 0.92–1.41, *P* = 0.22)

**Table 4.** Outcome and survival rates.

Outcome	Patients (*n* = 186)	Duration of treatment (days)
Complete resolution	107 (57.5)	167.2 ± 114.9, 144 (70–201)
Partial resolution	29 (15.5)	168.0 ± 59.8, 160 (119–217)
Recurrence	17 (9.1)	186.7 ± 111.1, 202.5 (103.5–270)
Death due to fungal sepsis	3 (1.6)	60.6 ± 42.6, 40 (36–124)
Death due to other reasons	30 (16.1)	64.6 ± 49.6, 45 (30–112)

Data are presented as means ± SD, medians and IQR, or as absolute frequencies (percentages) when appropriate.

## Discussion

Although osteoarticular infections due to *Aspergillus* spp. are relatively uncommon, they can present with increased frequency in patients with certain comorbidities such as immunodeficiency disorders. However, there are only small studies with proven osteoarticular aspergillosis that provide sufficient data regarding the diagnosis, treatment, and outcome of these infections. These small studies lack numerical power for any comparative outcomes. This is also reflected by the results of our systematic review, since 87% of the included studies were case reports, while only 2 studies included more than 5 patients. This systematic review reported on the outcomes of 186 patients with proven osteoarticular aspergillosis who received several different antifungal regimes with or without surgical treatment. Our results indicate that monotherapy is as effective as combination or sequential therapy, while voriconazole has similar efficacy with amphotericin B as monotherapy. Moreover, the better outcomes that were seen in surgically treated patients highlight the importance of surgical debridement of the infected focus for the successful treatment of osteoarticular aspergillosis.

The higher incidence of osteoarticular aspergillosis in immunocompromised patients was also confirmed by the results of our study. Specifically, 18.8% of the patients had chronic granulomatous disease, a genetic disorder that results in immunodeficiency, while 12.3% of them suffered from another immunodeficiency disorder or they were receiving immunosuppression therapy. In addition, hematologic neoplasia was reported in 16.1% of the included patients and corticosteroids in 17.7% of the patients. All these conditions are associated with immunodeficiency and susceptibility to fungal infections. Chronic granulomatous disease has been also identified by other studies as a predominant immunodeficiency disorder in patients with osteoarticular aspergillosis. Gamaletsou et al. conducted a systematic review evaluating 180 patients with proven or suspected osteoarticular aspegillosis and reported that chronic granulomatous disease was evident in 12.7% of all cases and in 73% of pediatric cases, while in our study chronic granulomatous disease was evident in even more patients (18.8% of all cases and 85% of pediatric cases [<16 years]).^156^

The most commonly isolated pathogen in our study was *A. fumigatus* (55.3%), followed by *A. flavus* (15.0%). Although this is similar to other studies in which *A. fumigatus* and *A. flavus* were also identified as the most common *Aspergillus* species, microbiology varied based on the age and the comorbidities of our population. *A. nidulans* was more frequently reported in younger patients, probably due to the higher incidence of primary immunodeficiency in this age group, while it was the most common pathogen in patients with chronic granulomatous disease (45%). This finding is consistent with the review study of Dotis et al. who reported on 46 cases of osteoarticular aspergillosis in patients with chronic granulomatous disease.^157^ Dotis et al. revealed that infections from *A. nidulans* were more common in these patients and were associated with higher mortality compared to other *Aspergillus* species.

Our results indicate that the route of infection varies depending on the location of the infection. Specifically, hematogenous spread was the most common route for spinal infection, while costal aspergillosis was associated with contiguous spread, and aspergillosis in lower and upper extremities was associated with direct inoculation following surgery or trauma. Hematogenous spread is the most common mechanism for both bacterial and fungal spondylodiscitis, while contiguous transpleural dissemination from a pulmonary focus is also commonly involved in spinal infections. Clinicians should consider the possibility of spinal aspergillosis in patients with pulmonary aspergillosis who complain of back pain, and should have a low threshold for ordering imaging studies to diagnose or rule out vertebral involvement. The predominance of contiguous spread in cases of costal aspergillosis also indicates direct extension from pulmonary foci due to the close proximity with these foci. Similar to spinal infections, further imaging evaluation with a CT scan (using bone window) or MRI is necessary in patients with pulmonary aspergillosis and persistent pleuritic pain, since these patients may also need surgical debridement in addition to the antifungal therapy for the successful eradication of the costal foci. As opposed to spinal and costal aspergillosis, direct inoculation was reported as the most common route in cases of infections in the upper and lower extremities, as well as in case of sternal aspergillosis. This highlights the importance of perioperative preventive measures in immunocompromised patients, while clinicians should consider these pathogens in case of posttraumatic or postoperative signs of infections in this population.

The high heterogeneity in the antifungal therapy for the treatment of osteoarticular aspergillosis was also reflected by the results of our study. Our results indicate that antifungal monotherapy has similar outcomes with antifungal combination/sequential therapy, while also amphotericin B monotherapy has similar outcomes with voriconazole monotherapy. The overall safety profile and pharmacokinetics of voriconazole are preferable than that of amphotericin B. This is also reflected by the Infectious Diseases Society of America (IDSA) guidelines which recommend voriconazole as the primary antifungal agent and liposomal amphotericin B as an alternative option for the treatment of osteoarticular aspergillosis.^158^ Amphotericin B is associated with significant nephrotoxicity, while the prolonged venous access for its administration is another significant disadvantage associated with certain complications such as thrombophlebitis and systemic infections. On the other hand, voriconazole has the flexibility of both parenteral and oral administration, and although it is also associated with certain adverse events such as solar hypersensitivity, it has minor nephrotoxicity and is considered a safer option compared to amphotericin B. Voriconazole has acceptable oral bioavailability (45–75% in children and 90% in adults), however, prolonged therapy can result in autoinduction of metabolism with decreasing levels.^159^ Therefore, monitoring of trough levels after establishment of initial therapeutic levels is recommended in patients who receive voriconazole to detect off-target trough levels due to autoinduction, which will require subsequent dose adjustments.^160^

The median duration of antifungal treatment was 5 months (155 days). Based on the IDSA guidelines, the recommended duration of therapy is at least 8 weeks–6 months.^158^ However, this recommendation is based upon small case series and expert opinion, while the wide range of the recommended duration also reflects the lack of high-quality evidence for the treatment of these infections. The minimum recommended duration of 60 days is in line only with the amphotericin B treatment as monotherapy (median duration 56 days in our study), while the median duration of all other antifungal regimes was significantly longer. The median duration of antifungal treatment in our study was also longer than the respective duration of medical treatment that was reported in the systematic review by Gamaletsou et al. (90 days), despite that fact that number of patients who received amphotericin B as monotherapy was comparable between the two studies.^156^ Prospective studies are needed to evaluate whether shorter antifungal therapies have comparable results with prolonged treatments. Last, we found that surgical debridement of the infected focus results in better outcomes in terms of infection eradication and is associated with lower relapse rate, which is consistent with the IDSA guidelines and the results of other studies that also highlight the importance of surgical treatment.^161^ However, the decision for surgical treatment should be based on several factors, such as patient's comorbidities and the location of the infection. The risk/benefit ratio of surgical treatment widely varies based on the location of the infection. The results of surgical treatment in terms of infection eradication are not the same for all locations. Moreover, surgery in certain locations is associated with higher morbidity than in other locations, since, for example, the risk of spine surgery is significantly higher than the risk of a surgical debridement of a toe infection.

Our systematic review is the largest review with biopsy proven *Aspergillus* spp. osteoarticular infection and extensive comparison of different treatment strategies. In our review, we identified 186 patients, while another systematic review by Gamaletsou et al. included a similar number of cases (180 patients).^156^ However, almost half of the patients in the review by Gamaletsou et al. were ‘probable’ cases and not confirmed cases by biopsy. Probable cases were patients with clinical and radiological signs indicative of osteomyelitis with positive culture of *Aspergillus* spp. from another location other than the bone, such as patients with pulmonary aspergillosis. However, the possibility of a bacterial and not *Apergillus* spp. osteomyelitis in these cases cannot be excluded, therefore, the true number of *Aspergillus* spp. osteoarticular infections in that review may be significantly lower. Moreover, our review is the first systematic review including only cases with full available data regarding significant parameters such as the treatment strategy (antifungal agents, surgical treatment) and the final outcome (complete vs. partial resolution, recurrence, death) of these infections, therefore, we eliminated any bias resulting from missing data. Last, we conducted a more comprehensive analysis of the collected data than any other systematic review, comparing surgical vs. conservative treatment, antifungal monotherapy vs. combination/sequential therapy, and amphotericin B vs. voriconazole in terms of infection resolution.

There are some limitations of this systematic review that must be also addressed. Due to publication bias, our results may reveal better treatment outcomes than the real success rate, since studies with positive results are more likely to be published. However, our strict inclusion criteria for studies with only detailed report of their outcomes minimize this risk, which is also shown by the fact that the rate of complete resolution of the infection in our study was lower than that reported in other recent systematic reviews. Moreover, the data that were analyzed for the comparisons of the different treatment approaches (antifungal agents, surgical treatment, monotherapy vs. combination therapy) are from small studies rather that high level large studies, therefore, the results of these comparisons should be interpreted with caution. However, the large number of included cases provides valuable information regarding the management of these infections. Another limitation is that the comparison of resolution rates between groups of patients receiving different therapeutic interventions is of observational nature and, thus, may have been affected by bias (confounding by indication). The treatment allocation was not randomized, and the indication of treatment might be related to the risk of outcomes; thus, the resulting imbalance in the underlying profiles between groups of patients receiving different treatments might generate biased results. Several factors such as the age and the underlying condition of the patients may affect the success rate of any treatment intervention. These confounding factors were not taken into consideration, and the comparison between the different treatment groups was not adjusted for these confounding factors. The comparison of the resolution rates between the different treatment modalities should be also interpreted with caution because the total follow up is not reported in many cases, therefore, estimations about the recurrence rate and the rate of complete/incomplete resolution can be biased by the heterogeneity in the duration of the follow up of the included patients. Last, the different time periods of the included studies is another potential bias, since different time periods are related to different diagnostic/treatment approaches and different antifungal agents.

In conclusion, osteoarticular aspergillosis should be considered in cases of immunocompromised patients with signs of infection and musculoskeletal symptoms such as low back pain or pleuritic pain. Prompt diagnosis of osteoarticular aspergillosis is essential since surgical debridement is crucial for its successful treatment as shown by the results of this review, whereas in bacterial infections the indications for surgical treatment can be more conservative. Moreover, the results of this study indicate that antifungal monotherapy is as effective as combination/sequential therapy, minimizing the risk of adverse events from antifungal agents. Last, voriconazole monotherapy have comparable results with amphotericin B monotherapy, and its favorable characteristics in terms of safety and route of administration makes voriconazole the recommended antifungal agent for the treatment of *Aspergillus* spp. osteoarticular infections. The duration of antifungal therapy highly varies in the literature, therefore, large prospective, carefully designed and adequately powered studies are warranted.in order to identify the optimal duration of antifungal therapy.

## Supplementary Material

myac052_Supplemental_Files

## References

[1] Henry MW , Miller AO, Walsh TJ, Brause BD. Fungal musculoskeletal infections. Infect Dis Clin North Am. 2017; 31 (2): 353–368. 28483045 10.1016/j.idc.2017.01.006

[2] Bariteau JT , Waryasz GR, McDonnell M et al. Fungal osteomyelitis and septic arthritis. J Am Acad Orthop Surg. 2014; 22 (6): 390–401. 24860135 10.5435/JAAOS-22-06-390

[3] Arastehfar A , Carvalho A, Houbraken J et al. *Aspergillus* fumigatus and aspergillosis: from basics to clinics. Stud Mycol. 2021; 100 (1): 100115. 34035866 10.1016/j.simyco.2021.100115 PMC8131930

[4] Koutserimpas C , Chamakioti I, Raptis K et al. Osseous infections caused by *Aspergillus* species. Diagnostics (Basel). 2022; 12 (1): 201. doi:10.3390/diagnostics12010201. 35054368 10.3390/diagnostics12010201 PMC8774735

[5] Gabrielli E , Fothergill AW, Brescini L et al. Osteomyelitis caused by *Aspergillus* species: a review of 310 reported cases. Clin Microbiol Infect. 2014; 20 (6): 559–565. 24303995 10.1111/1469-0691.12389

[6] Tsantes AG , Papadopoulos DV, Lytras T et al. Association of malnutrition with surgical site infection following spinal surgery: systematic review and meta-analysis. J Hosp Infect. 2020; 104 (1): 111–119. 31562915 10.1016/j.jhin.2019.09.015

[7] Tsantes AG , Papadopoulos DV, Vrioni G et al. Spinal infections: an update. Microorganisms. 2020; 8 (4): 476. 32230730 10.3390/microorganisms8040476 PMC7232330

[8] Abu Jawdeh L , Haidar R, Bitar F et al. *Aspergillus* vertebral osteomyelitis in a child with a primary monocyte killing defect: response to GM-CSF therapy. J Infect. 2000; 41 (1): 97–100. 11041713 10.1053/jinf.2000.0673

[9] Allen D , Ng S, Beaton K, Taussig D. Sternal osteomyelitis caused by *Aspergillus* fumigatus in a patient with previously treated Hodgkin's disease. J Clin Pathol. 2002; 55 (8): 616–618. 12147658 10.1136/jcp.55.8.616 PMC1769736

[10] AlMoosa ZA , AlFawaz T, AlFawaz F. Pott's puffy tumor due to *Aspergillus* fumigatus: a case report and review. Int J Pediatr Adolesc Med. 2016; 3 (3): 128–131. 30805483 10.1016/j.ijpam.2016.08.005 PMC6372439

[11] Al-Tawfiq JA , Al-Abdely HM. Vertebral osteomyelitis due to *Aspergillus* fumigatus in a patient with chronic granulomatous disease successfully treated with antifungal agents and interferon-gamma. Med Mycol. 2010; 48 (3): 537–541. 19886765 10.3109/13693780903325290

[12] Anderson J , Kron IL. Treatment of *Aspergillus* infection of the proximal aortic prosthetic graft with associated vertebral osteomyelitis. J Vasc Surg. 1984; 1 (4): 579–581. 6492301 10.1067/mva.1984.avs0010579

[13] Anjani G , Jindal AK, Prithvi A et al. *Aspergillus* fumigatus skull bone osteomyelitis and native valve endocarditis in a young boy: an unusual presentation of chronic granulomatous disease. J Clin Immunol. 2021; 41 (4): 814–816. 33452598 10.1007/s10875-020-00939-8

[14] Antkowiak TT , Polage CR, Wiedeman JA, Meehan JP, Jamali AA. Chondrolysis of the tibial plateau caused by articular aspergillosis after ACL autograft reconstruction: management with a fresh osteochondral allograft: a case report. J Bone Joint Surg Am. 2011; 93 (21): e124(1)–e124(6). 10.2106/JBJS.J.00782 22048103

[15] Asare KA , Jahng M, Pincus JL, Massie L, Lee SA. Sternal osteomyelitis caused by *Aspergillus* fumigatus following cardiac surgery: case and review. Med Mycol Case Rep. 2012; 2: 4–6. 24432203 10.1016/j.mmcr.2012.12.003 PMC3885922

[16] Assaad W , Nuchikat PS, Cohen L, Esguerra JV, Whittier FC. *Aspergillus* discitis with acute disc abscess. Spine (Phila Pa 1976). 1994; 19 (19): 2226–2229. 7809759 10.1097/00007632-199410000-00019

[17] Assaf A , Faure E, Sermet K et al. Successful treatment of *Aspergillus* fumigatus sternal osteomyelitis with isavuconazole in a heart transplant recipient. Transpl Infect Dis. 2020; 22 (5): e13313. 32386273 10.1111/tid.13313

[18] Austin KS , Testa NN, Luntz RK, Greene JB, Smiles S. *Aspergillus* infection of total knee arthroplasty presenting as a popliteal cyst. Case report and review of the literature. J Arthroplasty. 1992; 7 (3): 311–314. 1402949 10.1016/0883-5403(92)90055-u

[19] Aydin O , Bektas B, Aslan A, Yildirim AN, Arslan F. Voriconazole-treated *aspergillus* vertebral osteomyelitis in an immunocompetent patient. J Orthop Sci. 2020; doi:10.1016/j.jos.2020.09.003. 10.1016/j.jos.2020.09.003 33036828

[20] Ayhan AC , Ozkan K, Timur C, Aktas B, Ceyran AB. Chronic granulomatous *Aspergillus* synovitis: a case report. Mediterr J Hematol Infect Dis. 2013; 5 (1): e2013043. 23795281 10.4084/MJHID.2013.043 PMC3684320

[21] Babamahmoodi F , Shokohi T, Ahangarkani F, Nabili M, Alinezhad S. Rare case of *Aspergillus* ochraceus osteomyelitis of calcaneus bone in a patient with diabetic foot ulcers. Case Rep Med. 2015; 2015: 509827. 26064128 10.1155/2015/509827 PMC4443939

[22] Barnwell PA , Jelsma LF, Raff MJ. *Aspergillus* osteomyelitis. Report of a case and review of the literature. Diagn Microbiol Infect Dis. 1985; 3 (6): 515–519. 4064611 10.1016/s0732-8893(85)80008-0

[23] Bartash R , Guo Y, Pope JB et al. Periprosthetic hip joint infection with *Aspergillus* terreus: a clinical case and a review of the literature. Med Mycol Case Rep. 2017; 18: 24–27. 28808616 10.1016/j.mmcr.2017.07.006 PMC5544492

[24] Bathoorn E , Escobar Salazar N, Sepehrkhouy S et al. Involvement of the opportunistic pathogen *Aspergillus* tubingensis in osteomyelitis of the maxillary bone: a case report. BMC Infect Dis. 2013; 13 (1): 59. 23374883 10.1186/1471-2334-13-59 PMC3565948

[25] Batra S , Arora S, Meshram H et al. A rare etiology of cauda equina syndrome. J Infect Dev Ctries. 2011; 5 (1): 079–082. 10.3855/jidc.1001 21330747

[26] Beluffi G , Bernardo ME, Meloni G, Spinazzola A, Locatelli F. Spinal osteomyelitis due to *Aspergillus* flavus in a child: a rare complication after haematopoietic stem cell transplantation. Pediatr Radiol. 2008; 38 (6): 709–712. 18392819 10.1007/s00247-008-0789-x

[27] Bianchi R , Chekikian G, Ciboddo G et al. Primary sternal osteomyelitis by *Aspergillus* fumigatus. Br J Rheumatol. 1994; 33 (10): 994–995. 7921771 10.1093/rheumatology/33.10.994

[28] Bielorai B , Toren A, Wolach B et al. Successful treatment of invasive aspergillosis in chronic granulomatous disease by granulocyte transfusions followed by peripheral blood stem cell transplantation. Bone Marrow Transplant. 2000; 26 (9): 1025–1028. 11100285 10.1038/sj.bmt.1702651

[29] Bodur H , Ozoran K, Colpan A et al. Arthritis and osteomyelitis due to *Aspergillus* fumigatus: a 17 years old boy with chronic granulomatous disease. Ann Clin Microbiol Antimicrob. 2003; 2 (1): 2. 12605719 10.1186/1476-0711-2-2 PMC150380

[30] Brandt SJ , Thompson RL, Wenzel RP. Mycotic pseudoaneurysm of an aortic bypass graft and contiguous vertebral osteomyelitis due to *Aspergillus* fumigatus. Am J Med. 1985; 79 (2): 259–262. 3895911 10.1016/0002-9343(85)90019-1

[31] Bridwell KH , Campbell JW, Barenkamp SJ. Surgical treatment of hematogenous vertebral *Aspergillus* osteomyelitis. Spine (Phila Pa 1976). 1990; 15 (4): 281–285. 2191455 10.1097/00007632-199004000-00006

[32] Brodsky JW , Seidenfeld SM, Brooks B, Shabat S. *Aspergillus* osteomyelitis and lymphangitis in immunocompromised patient after toenail clipping. Foot Ankle Int. 2005; 26 (7): 576–578. 16045852 10.1177/107110070502600715

[33] Brown DL , Musher DM, Taffet GE. Hematogenously acquired *Aspergillus* vertebral osteomyelitis in seemingly immunocompetent drug addicts. West J Med. 1987; 147 (1): 84–85. 3321709 PMC1025835

[34] Bujak JS , Kwon-Chung KJ, Chusid MJ. Osteomyelitis and pneumonia in a boy with chronic granulomatous disease of childhood caused by a mutant strain of *Aspergillus* nidulans. Am J Clin Pathol. 1974; 61 (3): 361–367. 4131011 10.1093/ajcp/61.3.361

[35] Camargo JF , Seriburi V, Tenner M, El Khoury MY. *Aspergillus* osteomyelitis of the lumbar spine complicated with orbital apex syndrome: a potential role of the Batson's plexus in disease propagation. Med Mycol Case Rep. 2012; 1 (1): 9–12. 24371725 10.1016/j.mmcr.2012.02.004 PMC3854623

[36] Cartoni C , Capua A, Damico C, Potente G. *Aspergillus* osteomyelitis of the rib: sonographic diagnosis. J Clin Ultrasound. 1992; 20 (3): 217–220. 1313838 10.1002/jcu.1870200311

[37] Castelli C , Benazzo F, Minoli L et al. *Aspergillus* infection of the L3-L4 disc space in an immunosuppressed heart transplant patient. Spine (Phila Pa 1976). 1990; 15 (12): 1369–1373. 2281383 10.1097/00007632-199012000-00027

[38] Chang HM , Yu HH, Yang YH et al. Successful treatment of *Aspergillus* flavus spondylodiscitis with epidural abscess in a patient with chronic granulomatous disease. Pediatr Infect Dis J. 2012; 31 (1): 100–101. 21873929 10.1097/INF.0b013e3182309ec0

[39] Cimerman M , Gunde-Cimerman N, Zalar P, Perkovic T. Femur osteomyelitis due to a mixed fungal infection in a previously healthy man. J Clin Microbiol. 1999; 37 (5): 1532–1535. 10203517 10.1128/jcm.37.5.1532-1535.1999 PMC84821

[40] Collier J , Wolfe R, Lerner R, Nathan S, Mohsenifar Z. Spinal *aspergillus* abscess in a patient with bronchocentric granulomatosis. J Intensive Care Med. 1995; 10 (1): 45–48. 10155170 10.1177/088506669501000106

[41] Comacle P , Le Govic Y, Hoche-Delchet C et al. Spondylodiscitis due to *Aspergillus* terreus in an immunocompetent host: case report and literature review. Mycopathologia. 2016; 181 (7): 575–581. 27038797 10.1007/s11046-016-0007-6

[42] Cosgarea AJ , Tejani N, Jones JA. Carpal *Aspergillus* osteomyelitis: case report and review of the literature. J Hand Surg Am. 1993; 18 (4): 722–726. 8349990 10.1016/0363-5023(93)90327-Y

[43] Dai G , Wang T, Yin C et al. *Aspergillus* spondylitis: case series and literature review. BMC Musculoskelet Disord. 2020; 21 (1): 572. 32828133 10.1186/s12891-020-03582-x PMC7443290

[44] Dayan L , Sprecher H, Hananni A et al. *Aspergillus* vertebral osteomyelitis in chronic leukocyte leukemia patient diagnosed by a novel panfungal polymerase chain reaction method. Spine J. 2007; 7 (5): 615–617. 17905324 10.1016/j.spinee.2006.08.005

[45] De Bock R , Schrijvers D, Peetermans M. Pulmonary aspergillosis complicated by osteomyelitis. Acta Clin Belg. 1991; 46 (6): 397–400. 1665943 10.1080/17843286.1991.11718196

[46] De Vuyst D , Surmont I, Verhaegen J, Vanhaecke J. Tibial osteomyelitis due to *Aspergillus* flavus in a heart transplant patient. Infection. 1992; 20 (1): 48–49. 1314228 10.1007/BF01704898

[47] Diard F , Kozlowski K, Masel J, Marc J. Multifocal, chronic, nonstaphylococcal osteomyelitis in children (report of four cases–aspergillosis, Klebsiella, tuberculosis). Australas Radiol. 1983; 27 (1): 39–44. 6882301 10.1111/j.1440-1673.1983.tb02341.x

[48] Dotis J , Panagopoulou P, Filioti J et al. Femoral osteomyelitis due to *Aspergillus* nidulans in a patient with chronic granulomatous disease. Infection. 2003; 31 (2): 121–124. 12682820 10.1007/s15010-002-2167-1

[49] D'Sa S R , Singh S, Satyendra S, Mathews P. Case report of *Aspergillus* osteomyelitis of the ribs in an immunocompetent patient. J Glob Infect Dis. 2013; 5 (3): 118–120. 24049367 10.4103/0974-777X.116875 PMC3766334

[50] Dubbeld P , van Oostenbrugge RJ, Twinjstra A, Schouten HC. Spinal epidural abscess due to *Aspergillus* infection of the vertebrae: report of 3 cases. Neth J Med. 1996; 48 (1): 18–23. 8775748 10.1016/0300-2977(95)00048-8

[51] Elahi MM , Mitra A, Spears J, McClurken JB. Recalcitrant chest wall *Aspergillus* fumigatus osteomyelitis after coronary artery bypass grafting: successful radical surgical and medical management. Ann Thorac Surg. 2005; 79 (3): 1057–1059. 15734444 10.1016/j.athoracsur.2003.09.119

[52] Ersoy A , Akdag I, Akalin H, Sarisozen B, Ener B. Aspergillosis osteomyelitis and joint infection in a renal transplant recipient. Transplant Proc. 2007; 39 (5): 1662–1663. 17580212 10.1016/j.transproceed.2006.11.020

[53] Ersoy A , Dizdar OS, Koc AO, Akalin H, Ener B. *Aspergillus* fumigatus spondylodiskitis in renal transplant patient: voriconazole experience. Exp Clin Transplant. 2011; 9 (4): 265–269. 21819372

[54] Farhoudi A , Siadati A, Atarod L et al. Para vertebral abscess and rib osteomyelitis due to *Aspergillous* Fumigatus in a patient with Chronic Granulomatous Disease. Iran J Allergy Asthma Immunol. 2003; 2: 13–15. 17301351

[55] Figueres ML , Cantarovich D, Tattevin P et al. *Aspergillus* arthritis and organ transplantation. Clin Kidney J. 2012; 5 (3): 237–239. 26069774 10.1093/ckj/sfs024 PMC4400502

[56] Flynn PM , Magill HL, Jenkins JJ 3rd et al. *Aspergillus* osteomyelitis in a child treated for acute lymphoblastic leukemia. Pediatr Infect Dis J. 1990; 9 (10): 733–736. 2235147 10.1097/00006454-199010000-00010

[57] Gamaletsou MN , Rammaert B, Bueno MA et al. *Aspergillus* arthritis: analysis of clinical manifestations, diagnosis, and treatment of 31 reported cases. Med Mycol. 2017; 55 (3): 246–254. 27609563 10.1093/mmy/myw077 PMC6251616

[58] Garazzino S , Maiello A, DE Rosa FG, Aprato A, Di Perri G. Post-traumatic osteomyelitis due to *Aspergillus* flavus successfully treated with voriconazole: a case report. J Chemother. 2008; 20 (4): 524–526. 18676238 10.1179/joc.2008.20.4.524

[59] Gerlach UA , Kohler S, Sauer IM et al. *Aspergillus* spondylodiscitis after multivisceral transplantation. Ann Transplant. 2009; 14 (4): 52–57. 20009156

[60] Govender S , Kumar KP. *Aspergillus* spondylitis in immunocompetent patients. Int Orthop. 2001; 25 (2): 74–76. 11409455 10.1007/s002640000205 PMC3620633

[61] Grossman M. Aspergillosis of bone. Br J Radiol. 1975; 48 (565): 57–59. 1089024 10.1259/0007-1285-48-565-57

[62] Gupta PK , Mahapatra AK, Gaind R et al. *Aspergillus* spinal epidural abscess. Pediatr Neurosurg. 2001; 35 (1): 18–23. 11490186 10.1159/000050380

[63] Hall GL , Villanueva-Siles E, Borzykowski RM et al. *Aspergillus* osteomyelitis of the proximal humerus: a case report. Skeletal Radiol. 2012; 41 (8): 1021–1025. 22467007 10.1007/s00256-012-1401-x

[64] Hebert-Seropian S , Pelet S. *Aspergillus* Osteomyelitis of the scapula: a case report. JBJS Case Connect. 2020; 10 (1): e0343. 10.2106/JBJS.CC.19.00343 32044791

[65] Hendrix WC , Arruda LK, Platts-Mills TA et al. *Aspergillus* epidural abscess and cord compression in a patient with aspergilloma and empyema. Survival and response to high dose systemic amphotericin therapy. Am Rev Respir Dis. 1992; 145 (6): 1483–1486. 1596022 10.1164/ajrccm/145.6.1483

[66] Hodiamont CJ , Dolman KM, Ten Berge IJ et al. Multiple-azole-resistant *Aspergillus* fumigatus osteomyelitis in a patient with chronic granulomatous disease successfully treated with long-term oral posaconazole and surgery. Med Mycol. 2009; 47 (2): 217–220. 19101840 10.1080/13693780802545600

[67] Holmes PF , Osterman DW, Tullos HS. *Aspergillus* discitis. Report of two cases and review of the literature. Clin Orthop Relat Res. 1988; (226): 240–246. 3335098

[68] Horsburgh CR Jr. , Cannady PB Jr., Kirkpatrick CH. Treatment of fungal infections in the bones and joints with ketoconazole. J Infect Dis. 1983; 147 (6): 1064–1069. 6304204 10.1093/infdis/147.6.1064

[69] Hosalkar HS , Gill IP, Monsell F, Sau I, Ramsay A. Hip pain in a 12-year-old boy. Clin Orthop Relat Res. 2003; 416: 325–332. 10.1097/01.blo.0000093033.56370.02 14646776

[70] Hovi L , Saarinen UM, Donner U, Lindqvist C. Opportunistic osteomyelitis in the jaws of children on immunosuppressive chemotherapy. J Pediatr Hematol Oncol. 1996; 18 (1): 90–94. 8556379 10.1097/00043426-199602000-00018

[71] Jerome TJ , Kandasamy L, Terrence TK, Bhalaji S, Shanmugasundaram B. *Aspergillus* flavus infection of lower limb in an immune-competent patient. Cureus. 2020; 12 (7): e9342. 32850216 10.7759/cureus.9342 PMC7444893

[72] Jiang Z , Wang Y, Jiang Y, Xu Y, Meng B. Vertebral osteomyelitis and epidural abscess due to *Aspergillus* nidulans resulting in spinal cord compression: case report and literature review. J Int Med Res. 2013; 41 (2): 502–510. 23569017 10.1177/0300060513476432

[73] Kaneko J , Sugawara Y, Makuuchi M. *Aspergillus* osteomyelitis after liver transplantation. Liver Transpl. 2002; 8 (11): 1073–1075. 12424724 10.1053/jlts.2002.35778

[74] Karapinar B , Yilmaz D, Asar G, Vardar F. Disseminated invasive vertebral aspergillosis in an immunocompetent girl with a 7 year latent period. Pediatr Int. 2007; 49 (4): 516–518. 17587279 10.1111/j.1442-200X.2007.02412.x

[75] Karia S , Jeyapalan K, Kennedy B. *Aspergillus* fumigatus osteomyelitis in a patient receiving alemtuzumab for B-cell chronic lymphocytic leukaemia. Br J Haematol. 2011; 153 (2): 147. 21332707 10.1111/j.1365-2141.2011.08570.x

[76] Karthik K , Shetty AP, Rajasekaran S. Spontaneous cord transection due to invasive *aspergillus* spondylitis in an immunocompetent child. Eur Spine J. 2011; 20 (2): 188–192. 10.1007/s00586-010-1506-7 PMC3111527 20596734

[77] Khalid M , Ali SA. Fungal osteomyelitis in a patient with chronic granulomatous disease: case report and review of the literature. J Pak Med Assoc. 2018; 68 (9): 1387–1390. 30317271

[78] Kobayashi T , Lawler E, Samra H, Ford B, Sekar P. Prosthetic finger joint infection due to *Aspergillus* terreus. Open Forum Infect Dis. 2021; 8 (1): ofaa614. 33511236 10.1093/ofid/ofaa614 PMC7813175

[79] Korovessis P , Repanti M, Katsardis T, Stamatakis M. Anterior decompression and fusion for *Aspergillus* osteomyelitis of the lumbar spine associated with paraparesis. Spine (Phila Pa 1976). 1994; 19 (23): 2715–2718. 7899969

[80] Kumar M , Thilak J, Zahoor A, Jyothi A. Septic arthritis due to tubercular and *Aspergillus* co-infection. Indian J Orthop. 2016; 50 (3): 327–330. 27293296 10.4103/0019-5413.181783 PMC4885304

[81] Kwong CA , Puloski SK, Hildebrand KA. Fungal periprosthetic joint infection following total elbow arthroplasty: a case report and review of the literature. J Med Case Rep. 2017; 11 (1): 20. 28109195 10.1186/s13256-016-1176-0 PMC5251295

[82] Landaburu MF , Lopez Daneri G, Ploszaj F et al. Osteomyelitis of the rib cage by *Aspergillus* flavus. Rev Iberoam Micol. 2019; 36 (2): 86–89. 31128846 10.1016/j.riam.2019.02.002

[83] Li XF , Liu ZD, Xia Q et al. Primary *Aspergillus* spondylodiscitis in a liver transplant recipient. Chin Med J (Engl). 2012; 125 (15): 2772–2774. 22931991

[84] Liu JW , Liu PY, Lai KL. *Aspergillus* terreus septic arthritis and pyomyositis of shoulder. J Formos Med Assoc. 2021; 120 (4): 1153–1154. 33115625 10.1016/j.jfma.2020.10.017

[85] Lodge BA , Ashley ED, Steele MP, Perfect JR. *Aspergillus* fumigatus empyema, arthritis, and calcaneal osteomyelitis in a lung transplant patient successfully treated with posaconazole. J Clin Microbiol. 2004; 42 (3): 1376–1378. 15004125 10.1128/JCM.42.3.1376-1378.2004 PMC356879

[86] Lyons MK , Neal MT, Patel NP, Vikram HR. Progressive back pain due to *aspergillus* nidulans vertebral osteomyelitis in an immunocompetent patient: surgical and antifungal management. Case Rep Orthop. 2019; 2019: 4268468. 31355034 10.1155/2019/4268468 PMC6636486

[87] Ma H , Kuang L, Lv G, Wang B, Lian Z. Vertebral aspergillosis in a patient with autosomal-dominant hyper-IgE syndrome. Clin Vaccine Immunol. 2014; 21 (1): 107–109. 24197892 10.1128/CVI.00529-13 PMC3910922

[88] Mamishi S , Zomorodian K, Saadat F et al. A case of invasive aspergillosis in CGD patient successfully treated with Amphotericin B and INF-gamma. Ann Clin Microbiol Antimicrob. 2005; 4 (1): 4. 15745454 10.1186/1476-0711-4-4 PMC555544

[89] Mamishi S , Zomorodian K, Saadat F, Jalali SZ, Geramishoar M. Osteomyelitis and lung abscess due to *Aspergillus* fumigatus in a chronic granulomatous disease patient. Curr Med Mycol. 2016; 2 (3): 37–41. 10.18869/acadpub.cmm.2.3.37 PMC5490289 28681028

[90] Martinez M , Lee AS, Hellinger WC, Kaplan J. Vertebral *Aspergillus* osteomyelitis and acute diskitis in patients with chronic obstructive pulmonary disease. Mayo Clin Proc. 1999; 74 (6): 579–583. 10377933 10.4065/74.6.579

[91] Mawk JR , Erickson DL, Chou SN, Seljeskog EL. Aspergillus infections of the lumbar disc spaces. Report of three cases. J Neurosurg. 1983; 58 (2): 270–274. 6848687 10.3171/jns.1983.58.2.0270

[92] McCaslin AF , Lall RR, Wong AP et al. Thoracic spinal cord intramedullary *aspergillus* invasion and abscess. J Clin Neurosci. 2015; 22 (2): 404–406. 25088481 10.1016/j.jocn.2014.04.030

[93] McGregor A , McNicol D, Collignon P. *Aspergillus*-induced discitis. A role for itraconazole in therapy? Spine (Phila Pa 1976). 1992; 17 (12): 1512–1514. 1335170

[94] Mekan SF , Saeed O, Khan JA. Invasive aspergillosis with polyarthritis. Mycoses. 2004; 47 (11–12): 518–520. 15601460 10.1111/j.1439-0507.2004.01031.x

[95] Menon A , Rodrigues C, Soman R, Sunavala A, Agashe VM. *Aspergillus* osteomyelitis of the ribs in immunocompetent hosts: report of two rare cases. J Orthop Case Rep. 2017; 7 (4): 61–64. 10.13107/jocr.2250-0685.854 PMC5702709 29181357

[96] Mirhosseini SJ , Salehi M, Ali-Hassan-Sayegh S, Forouzannia K, Karimi-Bondarabadi AA. Costochondritis caused by *Aspergillus* flavus following cardiac surgery. Acta Med Iran. 2013; 51 (10): 733–735. 24338151

[97] Mohammadpour M , Mamishi S, Oaji M, Pourpak Z, Parvaneh N. Successful treatment of fungal osteomyelitis with voriconazole in a patient with chronic granulomatous disease. Iran J Pediatr. 2010; 20 (4): 487–490. 23056752 PMC3446094

[98] Morgenlander JC , Rossitch E Jr., Rawlings CE 3rd. *Aspergillus* disc space infection: case report and review of the literature. Neurosurgery. 1989; 25 (1): 126–129. 2666879

[99] Nasca RJ , McElvein RB. *Aspergillus* fumigatus osteomyelitis of the thoracic spine treated by excision and interbody fusion. Spine (Phila Pa 1976). 1985; 10 (9): 848–850. 3911444 10.1097/00007632-198511000-00013

[100] Natesan S , Abraham G, Mathew M, Lalitha MK, Srinivasan CN. Secondary sternal *Aspergillus* osteomyelitis in a diabetic hemodialysis patient with previous allograft rejection. Hemodial Int. 2007; 11 (4): 403–405. 17922735 10.1111/j.1542-4758.2007.00208.x

[101] Nicholson S , King R, Chumas P, Russell J, Liddington M. *Aspergillus* Osteomyelitis of the Skull. J Craniofac Surg. 2016; 27 (5): e504–e506. 27391523 10.1097/SCS.0000000000002797

[102] Oh IS , Seo JY, Ha KY, Kim YC. Treatment for multiple *Aspergillus* spondylitis including a hip joint. Asian Spine J. 2009; 3 (2): 106–112. 20404956 10.4184/asj.2009.3.2.106 PMC2852076

[103] Ordaya EE , Johnson JR, Drekonja DM, Niehans GE, Kaka AS. *Aspergillus* osteomyelitis secondary to chronic necrotizing pulmonary aspergillosis in a patient with rheumatoid arthritis. Cureus. 2021; 13 (9): e17774. 34659985 10.7759/cureus.17774 PMC8494381

[104] Panigrahi S , Nagler A, Or R et al. Indolent *aspergillus* arthritis complicating fludarabine-based non-myeloablative stem cell transplantation. Bone Marrow Transplant. 2001; 27 (6): 659–661. 11319599 10.1038/sj.bmt.1702853

[105] Park KU , Lee HS, Kim CJ, Kim EC. Fungal discitis due to *Aspergillus* terreus in a patient with acute lymphoblastic leukemia. J Korean Med Sci. 2000; 15 (6): 704–707. 11194199 10.3346/jkms.2000.15.6.704 PMC3054694

[106] Park SB , Kang MJ, Whang EA, Han SY, Kim HC. A case of fungal sepsis due to *aspergillus* spondylitis followed by cytomegalovirus infection in a renal transplant recipient. Transplant Proc. 2004; 36 (7): 2154–2155. 15518782 10.1016/j.transproceed.2004.08.049

[107] Pattanashetty OB , Dayanand BB, Bhavi SB, Bami M. Rare case of isolated *Aspergillus* osteomyelitis of Toe: presentation and management. J Orthop Case Rep. 2013; 3 (2): 29–31. 10.13107/jocr.2250-0685.098 PMC4719239 27298903

[108] Pattison RM , O'Donnell MA, Powles R, Moss AL. A painful knee in an immunocompromised patient. Postgrad Med J. 1997; 73 (855): 49–50. 9039414 10.1136/pgmj.73.855.49 PMC2431181

[109] Peters-Christodoulou MN , de Beer FC, Bots GT et al. Treatment of postoperative *Aspergillus* fumigatus spondylodiscitis with itraconazole. Scand J Infect Dis. 1991; 23 (3): 373–376. 1652789 10.3109/00365549109024325

[110] Radhakrishnan S , Mujeeb H, Radhakrishnan C. Central skull base osteomyelitis secondary to invasive *aspergillus* sphenoid sinusitis presenting with isolated 12th nerve palsy. IDCases. 2020; 22: e00930. 32923366 10.1016/j.idcr.2020.e00930 PMC7475189

[111] Raj KA , Srinivasamurthy BC, Nagarajan K, Sinduja MG. A rare case of spontaneous *Aspergillus* spondylodiscitis with epidural abscess in a 45-year-old immunocompetent female. J Craniovertebr Junction Spine. 2013; 4 (2): 82–84. 24744568 10.4103/0974-8237.128538 PMC3980562

[112] Rassa M. Vertebral aspergillosis with preservation of the disc. Br J Radiol. 1977; 50 (600): 918–920. 338084 10.1259/0007-1285-50-600-918

[113] Redmond A , Carre IJ, Biggart JD, Mackenzie DW. Aspergillosis (*Aspergillus Nidulans*) involving bone. J Pathol Bacteriol. 1965; 89 (1): 391–395. 14263488 10.1002/path.1700890147

[114] Richards RH , Priaulx LR. A case of *Aspergillus* osteomyelitis complicating an open fracture of the tibia. Injury. 1988; 19 (2): 129–130. 3058608 10.1016/0020-1383(88)90094-0

[115] Rodriguez-Hernandez MJ , Jimenez-Mejias ME, Montero JM, Regordan C, Ferreras G. *Aspergillus* fumigatus cranial infection after accidental traumatism. Eur J Clin Microbiol Infect Dis. 2001; 20 (9): 655–656. 11714049 10.1007/s100960100579

[116] Roselle GA , Baird IM. *Aspergillus* flavipes group osteomyelitis. Arch Intern Med. 1979; 139 (5): 590–592. 443955

[117] Routray C , Nwaigwe C. Sternal osteomyelitis secondary to *Aspergillus* fumigatus after cardiothoracic surgery. Med Mycol Case Rep. 2020; 28: 16–19. 32274324 10.1016/j.mmcr.2020.03.003 PMC7132061

[118] Saba R , Bekoz H, Karadogan I et al. Septic arthritis due to *Aspergillus* treated with amphotericin B lipid complex and surgical debridement. J Chemother. 2004; 16 (2): 218–220. 15216961 10.1179/joc.2004.16.2.218

[119] Sabo MC , Blain M, McCulloch D et al. Back pain in a 23-Year-Old male with X-Linked chronic granulomatous disease. Open Forum Infect Dis. 2019; 6 (11): ofz449. 31723573 10.1093/ofid/ofz449 PMC6834088

[120] Sachs MK , Paluzzi RG, Moore JH Jr., Fraimow HS, Ost D. Amphotericin-resistant *aspergillus* osteomyelitis controlled by itraconazole. Lancet. 1990; 335 (8703): 1475. 10.1016/0140-6736(90)91513-a 1972255

[121] Salloum A , Rao S, Havasi A, Miljkovic G, Amoateng-Adjepong Y. *Aspergillus* rib and vertebral osteomyelitis in a former intravenous drug user. Am J Med. 2004; 116 (3): 208–209. 14749170 10.1016/j.amjmed.2003.05.006

[122] Seligsohn R , Rippon JW, Lerner SA. *Aspergillus* terreus osteomyelitis. Arch Intern Med. 1977; 137 (7): 918–920. 879933

[123] Senosain-Leon V , Hidalgo-Benites A, Arriola-Montenegro J, D'Angelo-Piaggio L, Beas R. Invasive pulmonary aspergillosis with *Aspergillus* vertebral osteomyelitis in an HIV-infected adult: a case report. Int J STD AIDS. 2019; 30 (11): 1140–1142. 31558121 10.1177/0956462419865403

[124] Sethi S , Siraj F, Kalra K, Chopra P. *Aspergillus* vertebral osteomyelitis in immunocompetent patients. Indian J Orthop. 2012; 46 (2): 246–250. 22448068 10.4103/0019-5413.93693 PMC3308671

[125] Shashidhar N , Tripathy SK, Balasubramanian S, Dhanakodi N, Venkataramaiah S. *Aspergillus* spondylodiscitis in an immunocompetent patient following spinal anesthesia. Orthop Surg. 2014; 6 (1): 72–77. 24590999 10.1111/os.12091 PMC6583265

[126] Sohn YJ , Yun JH, Yun KW et al. *Aspergillus* terreus spondylodiscitis in an immunocompromised child. Pediatr Infect Dis J. 2019; 38 (2): 161–163. 29912843 10.1097/INF.0000000000002125

[127] Sonin AH , Stern SH, Levi E. Primary *Aspergillus* osteomyelitis in the tibia of an immunosuppressed man. AJR Am J Roentgenol. 1996; 166 (6): 1277–1279. 8633431 10.2214/ajr.166.6.8633431

[128] Soto-Hurtado EJ , Marin-Gamez E, Segura-Dominguez N, Jimenez-Onate F. Pleural aspergillosis with bronchopleurocutaneous fistula and costal bone destruction: a case report. Lung. 2005; 183 (6): 417–423. 16465601 10.1007/s00408-005-2553-4

[129] Steinfeld S , Durez P, Hauzeur JP, Motte S, Appelboom T. Articular aspergillosis: two case reports and review of the literature. Br J Rheumatol. 1997; 36 (12): 1331–1334. 9448597 10.1093/rheumatology/36.12.1331

[130] Stodulski D , Kowalska B, Stankiewicz C. Otogenic skull base osteomyelitis caused by invasive fungal infection. Case report and literature review. Eur Arch Otorhinolaryngol. 2006; 263 (12): 1070–1076. 16896755 10.1007/s00405-006-0118-7

[131] Sun L , Zhang L, Wang K, Wang W, Tian M. Fungal osteomyelitis after arthroscopic anterior cruciate ligament reconstruction: a case report with review of the literature. Knee. 2012; 19 (5): 728–731. 22209694 10.1016/j.knee.2011.10.007

[132] Tack KJ , Rhame FS, Brown B, Thompson RC Jr., *Aspergillus* osteomyelitis. Report of four cases and review of the literature. Am J Med. 1982; 73 (2): 295–300. 7114084 10.1016/0002-9343(82)90192-9

[133] Takagi Y , Yamada H, Ebara H et al. *Aspergillus* terreus spondylodiscitis following an abdominal stab wound: a case report. J Med Case Rep. 2019; 13 (1): 172. 31164170 10.1186/s13256-019-2109-5 PMC6549268

[134] Tang TJ , Janssen HL, van der Vlies CH et al. *Aspergillus* osteomyelitis after liver transplantation: conservative or surgical treatment? Eur J Gastroenterol Hepatol. 2000; 12 (1): 123–126. 10656222 10.1097/00042737-200012010-00022

[135] Tavakoli M , Hedayati MT, Mirhendi H et al. The first rare and fatal case of invasive aspergillosis of spinal cord due to *Aspergillus* nidulans in an Iranian child with chronic granulomatosis disease: review of literature. Curr Med Mycol. 2020; 6 (1): 55–60. 32420510 10.18502/cmm.6.1.2551 PMC7217256

[136] Tew CW , Han FC, Jureen R, Tey BH. *Aspergillus* vertebral osteomyelitis and epidural abscess. Singapore Med J. 2009; 50 (4): e151–4. 19421672

[137] Tiwari V , Khatri K, Khan SA, Nath D. Disseminated *Aspergillus* flavus following septic arthritis in an immunocompetent patient: a case report. BMC Res Notes. 2014; 7 (1): 709. 25301635 10.1186/1756-0500-7-709 PMC4200168

[138] Tsumura N , Akasu Y, Yamane H et al. *Aspergillus* osteomyelitis in a child who has p67-phox-deficient chronic granulomatous disease. Kurume Med J. 1999; 46 (1): 87–90. 10319618 10.2739/kurumemedj.46.87

[139] Uehara Y , Kasai H, Nakajima T, Tanabe N, Tatsumi K, Yoshino I. *Aspergillus* sternomyelitis developed from Chronic Pulmonary Aspergillosis as a late complication to lobectomy for lung cancer. Intern Med. 2018; 57 (20): 2991–2994. 29877261 10.2169/internalmedicine.0334-17 PMC6232016

[140] Vaishya S , Sharma MS. Spinal *Aspergillus* vertebral osteomyelitis with extradural abscess: case report and review of literature. Surg Neurol. 2004; 61 (6): 551–555; discussion 555. 15165794 10.1016/j.surneu.2003.06.005

[141] van Ooij A , Beckers JM, Herpers MJ, Walenkamp GH. Surgical treatment of *aspergillus* spondylodiscitis. Eur Spine J. 2000; 9 (1): 75–79. 10766082 10.1007/s005860050014 PMC3611357

[142] van't Wout JW , Raven EJ, van der Meer JW. Treatment of invasive aspergillosis with itraconazole in a patient with chronic granulomatous disease. J Infect. 1990; 20 (2): 147–150. 2156938 10.1016/0163-4453(90)93418-r

[143] Verghese S , Chellamma T, Cherian KM. Osteomyelitis of the rib caused by *Aspergillus* flavus following cardiac surgery. Mycoses. 2009; 52 (1): 91–93. 18522699 10.1111/j.1439-0507.2008.01541.x

[144] Vinas FC , King PK, Diaz FG. Spinal *aspergillus* osteomyelitis. Clin Infect Dis. 1999; 28 (6): 1223–1229. 10451157 10.1086/514774

[145] Vlasveld LT , Delemarre JF, Beynen JH, Rodenhuis S. Invasive aspergillosis complicated by subclavian artery occlusion and costal osteomyelitis after autologous bone marrow transplantation. Thorax. 1992; 47 (2): 136–137. 1549823 10.1136/thx.47.2.136 PMC463600

[146] Wagner DK , Varkey B, Sheth NK, DaMert GJ. Epidural abscess, vertebral destruction, and paraplegia caused by extending infection from an aspergilloma. Am J Med. 1985; 78 (3): 518–522. 3976709 10.1016/0002-9343(85)90349-3

[147] Wang L , Liu L, Song Y. A rare case of thoracolumbar aspergillosis involving T10-L5. Spine J. 2015; 15 (9): 2110–2111. 25982435 10.1016/j.spinee.2015.05.007

[148] Watanabe C , Yajima S, Taguchi T et al. Successful unrelated bone marrow transplantation for a patient with chronic granulomatous disease and associated resistant pneumonitis and *Aspergillus* osteomyelitis. Bone Marrow Transplant. 2001; 28 (1): 83–87. 11498749 10.1038/sj.bmt.1703086

[149] Weclawiak H , Garrouste C, Kamar N et al. *Aspergillus* fumigatus-related spondylodiscitis in a heart transplant patient successfully treated with voriconazole. Transplant Proc. 2007; 39 (8): 2627–2628. 17954195 10.1016/j.transproceed.2007.08.014

[150] Williams RL , Fukui MB, Meltzer CC et al. Fungal spinal osteomyelitis in the immunocompromised patient: MR findings in three cases. AJNR Am J Neuroradiol. 1999; 20 (3): 381–385. 10219401 PMC7056067

[151] Winslow CP , Dichard A, McGuire KA. Osteomyelitis of the temporomandibular joint. Am J Otolaryngol. 2001; 22 (2): 142–145. 11283831 10.1053/ajot.2001.22577

[152] Yang H , Shah AA, Nelson SB, Schwab JH. Fungal spinal epidural abscess: a case series of nine patients. Spine J. 2019; 19 (3): 516–522. 30121322 10.1016/j.spinee.2018.08.001

[153] Yoon KW , Kim YJ. Lumbar *Aspergillus* osteomyelitis mimicking pyogenic osteomyelitis in an immunocompetent adult. Br J Neurosurg. 2015; 29 (2): 277–279. 25221965 10.3109/02688697.2014.957648

[154] Yoon PW , Song JH, Yoon KS et al. *Aspergillus* septic arthritis of the hip in an immunocompetent middle-aged female with undiagnosed recurrent pulmonary aspergillosis. Hip Pelvis. 2015; 27 (3): 196–200. 27536626 10.5371/hp.2015.27.3.196 PMC4972727

[155] Zhu LP , Chen XS, Wu JQ, Yang FF, Weng XH. *Aspergillus* vertebral osteomyelitis and ureteral obstruction after liver transplantation. Transpl Infect Dis. 2011; 13 (2): 192–199. 21457422 10.1111/j.1399-3062.2011.00599.x

[156] Gamaletsou MN , Rammaert B, Bueno MA et al. *Aspergillus* osteomyelitis: epidemiology, clinical manifestations, management, and outcome. J Infect. 2014; 68 (5): 478–493. 24378282 10.1016/j.jinf.2013.12.008 PMC4214682

[157] Dotis J , Roilides E. Osteomyelitis due to *Aspergillus* species in chronic granulomatous disease: an update of the literature. Mycoses. 2011; 54 (6): e686–e696. 21615532 10.1111/j.1439-0507.2010.02001.x

[158] Patterson TF , Thompson GR 3rd, Denning DW et al. Practice guidelines for the diagnosis and management of aspergillosis: 2016 update by the infectious diseases society of America. Clin Infect Dis. 2016; 63 (4): e1–e60. 27365388 10.1093/cid/ciw326 PMC4967602

[159] Neely M , Rushing T, Kovacs A, Jelliffe R, Hoffman J. Voriconazole pharmacokinetics and pharmacodynamics in children. Clin Infect Dis. 2010; 50 (1): 27–36. 19951112 10.1086/648679 PMC2803104

[160] Hu L , Dai TT, Zou L et al. Therapeutic drug monitoring of voriconazole in children from a tertiary care center in China. Antimicrob Agents Chemother. 2018; 62 (12): e00955–18. doi:10.1128/AAC.00955-18. 30150475 10.1128/AAC.00955-18 PMC6256783

[161] Koehler P , Tacke D, Cornely OA. *Aspergillosis* of bones and joints—a review from 2002 until today. Mycoses. 2014; 57 (6): 323–335. 24397460 10.1111/myc.12165

