# Supplementary material for: Aspergillus spp. osteoarticular infections: an updated systematic review on the diagnosis, treatment and outcomes of 186 confirmed cases
Source: Med Mycol. 2022 Jul 22;60(8):myac052. doi: 10.1093/mmy/myac052 (PMC9849853; doi:10.1093/mmy/myac052)
Supplement: myac052_Supplemental_Files [file myac052_supplemental_files.zip › mm-2022-0098-File005.docx]

Supplementary Table 2. Commonly used antifungal agents and dosage.

| Antifungal agents | Patients (n=186) | Dose (mg)* |
| --- | --- | --- |
| Amphotericin B | 114 (61.2) | - |
| Voriconazole | 61 (32.7) | 440.7 ± 120.5,  400.0 (380.0-460.0)/ per day |
| Itraconazole | 48 (25.8) | 385.7 ± 37.7,  400.0 (400.0-400.0)/ per day |
| 5-Fluorocytosine | 21 (11.2) | - |
| *Footnote:* Data are presented as means ± SD, medians and interquartile ranges (IQR), or as absolute frequencies (percentages) when appropriate.  *Dose of Amphotericin was not calculated due to the different forms of amphotericin B that were administered to the included patients | | |
