# Supplementary material for: Aspergillus spp. osteoarticular infections: an updated systematic review on the diagnosis, treatment and outcomes of 186 confirmed cases
Source: Med Mycol. 2022 Jul 22;60(8):myac052. doi: 10.1093/mmy/myac052 (PMC9849853; doi:10.1093/mmy/myac052)
Supplement: myac052_Supplemental_Files [file myac052_supplemental_files.zip › mm-2022-0098-File004.docx]

Supplementary Table 1. Anatomical distribution and route of infection

| Location | Infected foci (n=192) | | Hematogenous spread* | Contiguous spread* | Direct inoculation* |
| --- | --- | --- | --- | --- | --- |
|  | As a single focus (n=180) | As one of two foci  (n=12) |  |  |  |
| Spine | 95 (49.4) | 5 (2.6) | 42 (42.0) | 34 (34.0) | 24 (24.0) |
| Ribs and sternum | 33 (17.1) | 2 (1.0) | 2 (5.7) | 25 (71.4) | 8 (22.8) |
| Long bones lower extremity | 8 (4.1) | 1 (0.5) | 3 (33.3) | 3 (33.3) | 3 (33.3) |
| Cranial bones | 8 (4.1) | 1 (0.5) | 4 (44.4) | 3 (33.3) | 2 (22.2) |
| Maxilla or mandible | 5 (2.6) | 0 (0.0) | 0 (0.0) | 1 (20.0) | 4 (80.0) |
| Foot | 6 (3.1) | 0 (0.0) | 2 (33.3) | 0 (0.0) | 4 (66.6) |
| Long bones upper extremity | 2 (1.0) | 1 (0.5) | 0 (0.0) | 0 (0.0) | 3 (100.0) |
| Pelvis (iliac, sacral) | 0 (0.0) | 2 (1.0) | 1 (50.0 | 0 (0.0) | 1 (50.0 |
| Scapula | 1 (0.5) | 0 (0.0) | 0 (0.0) | 0 (0.0) | 1 (100.0) |
| Septic arthritis  Elbow or shoulder  Hip  Knee  Ankle | 3 (1.5)  6 (3.1)  10 (5.2)  3 (1.5) | 0 (0.0)  0 (0.0)  0 (0.0)  0 (0.0) | 0 (0.0)  0 (0.0)  1 (10.0)  1 (33.3) | 0 (0.0)  2 (33.3)  3 (30.0)  1 (33.3) | 3 (100.0)  4 (66.6)  6 (60.0)  1 (33.3) |
| Total | 192 (100) | | 56 (29.1) | 72 (37.5) | 64 (33.3) |
| *Footnote:* Data are presented as absolute frequencies (percentages).  * The percentages are calculated per location | | | | | |
